# Efficacy of Fenfluramine and Norfenfluramine Enantiomers and Various Antiepileptic Drugs in a Zebrafish Model of Dravet Syndrome

**DOI:** 10.1007/s11064-021-03358-2

**Published:** 2021-05-26

**Authors:** Jing Li, Maxim Nelis, Jo Sourbron, Daniëlle Copmans, Lieven Lagae, Deirdre Cabooter, Peter A. M. de Witte

**Affiliations:** 1 Department of Pharmaceutical and Pharmacological Sciences, Laboratory for Molecular Biodiscovery, University of Leuven (KU Leuven), Herestraat 49, 824, 3000 Leuven, Belgium; 2 Department of Pharmaceutical and Pharmacological Sciences, Laboratory for Pharmaceutical Analysis, University of Leuven (KU Leuven), Leuven, Belgium; 3 Department of Pediatric Neurology, University Hospitals Leuven, Leuven, Belgium

**Keywords:** Dravet syndrome, Zebrafish, Antiepileptic activity, Fenfluramine, Norfenfluramine, Enantiomers

## Abstract

Dravet syndrome (DS) is a rare genetic encephalopathy that is characterized by severe seizures and highly resistant to commonly used antiepileptic drugs (AEDs). In 2020, FDA has approved fenfluramine (FFA) for treatment of seizures associated with DS. However, the clinically used FFA is a racemic mixture (i.e. (±)-FFA), that is substantially metabolized to norfenfluramine (norFFA), and it is presently not known whether the efficacy of FFA is due to a single enantiomer of FFA, or to both, and whether the norFFA enantiomers also contribute significantly. In this study, the antiepileptic activity of enantiomers of FFA (i.e. (+)-FFA and (−)-FFA) and norFFA (i.e. (+)-norFFA and (−)-norFFA) was explored using the zebrafish *scn1Lab*^*−/−*^ mutant model of DS. To validate the experimental conditions used, we assessed the activity of various AEDs typically used in the fight against DS, including combination therapy. Overall, our results are highly consistent with the treatment algorithm proposed by the updated current practice in the clinical management of DS. Our results show that (+)-FFA, (−)-FFA and (+)-norFFA displayed significant antiepileptic effects in the preclinical model, and thus can be considered as compounds actively contributing to the clinical efficacy of FFA. In case of (−)-norFFA, the results were less conclusive. We also investigated the uptake kinetics of the enantiomers of FFA and norFFA in larval zebrafish heads. The data show that the total uptake of each compound increased in a time-dependent fashion. A somewhat similar uptake was observed for the (+)-norFFA and (−)-norFFA, implying that the levo/dextrotation of the structure did not dramatically affect the uptake. Significantly, when comparing (+)-FFA with the less lipophilic (+)-norFFA, the data clearly show that the nor-metabolite of FFA is taken up less than the parent compound.

## Introduction

Dravet syndrome (DS) is a rare, but severe developmental epileptic encephalopathy that begins in infancy [1, 2]. The first seizures are typically triggered by fever, and are characterized by long-lasting hemiclonic or generalized clonic or tonic–clonic convulsions. Later, the seizures evolve with age, and multiple seizure types may occur, such as focal, atypical absences, and myoclonic seizures. Furthermore, motor dysfunction, behavioural disorder, and cognitive impairment appear [3, 4]. Also increased incidence of mortality is reported in DS patients, especially due to a higher risk of sudden unexpected death [5, 6]. Regarding the genetic architecture of DS, a de novo mutation in the gene *SCN1A* which encodes for an α (pore-forming) subunit of the brain voltage gated sodium channel type-1 (Na_V_1.1), occurs in a large majority of patients [7].

Most DS patients are highly resistant to treatment with commonly used antiepileptic drugs (AEDs). For instance, around 45% of DS patients in Europe experienced on average more than four tonic–clonic seizures per month, even when treated with polytherapy regimens [8]. Algorithms for management of DS have been proposed by experts in North America [9] and Europe [10] to optimize the treatment outcome of DS patients with a minimal risk for toxicity. The most recent flowchart proposed by Cross and coworkers consists of valproate (VPA) as a first line treatment [10]. When a clear DS diagnosis is given and seizures continue, stiripentol (STP) [with or without clobazam (CLB)] or cannabidiol (CBD) or fenfluramine (FFA) can be added. As an alternative, the administration of topiramate (TPM) or a ketogenic diet may be considered [10].

In 2020, FDA approved FFA for treatment of seizures associated with DS. As a potent releaser and reuptake inhibitor for 5-hydroxytryptamine (5-HT, serotonin), FFA was initially used as an anorectic in polytherapy with phentermine, but was withdrawn from the market in 1997 due to cardiopulmonary side effects at high dosages [10]. However, the successful use of low dosage FFA as an add-on therapy for the treatment of DS was reported by Ceulemans et al. [11, 12] including the achievement of seizure free cases. Later clinical trials have further confirmed the efficacy and safety of FFA in treatment of DS. Importantly, no cardiovascular adverse effects were observed in these trials [1].

**Fig. 1:**
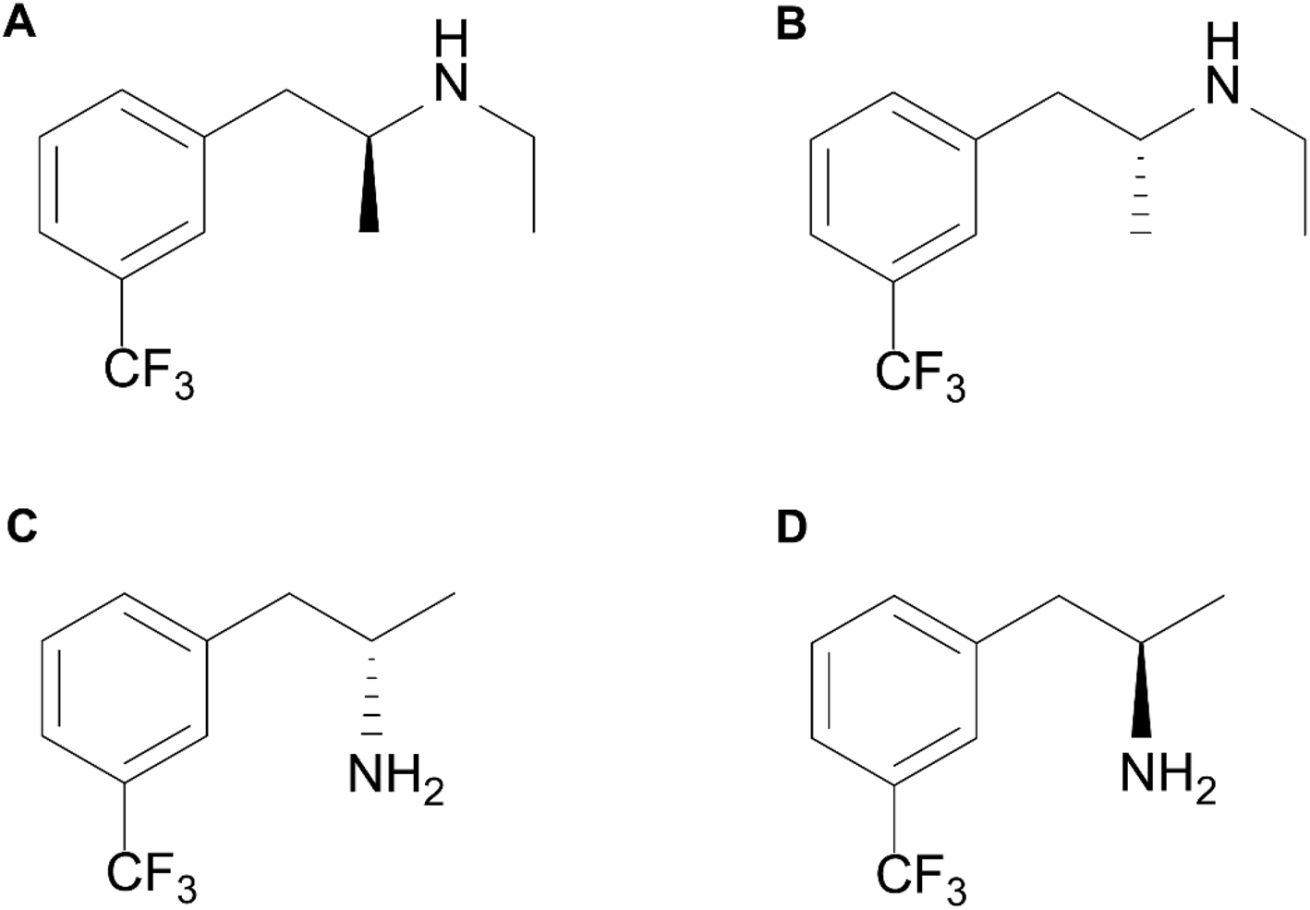
The structure of (+)-FFA (**A**), (-)-FFA (**B**), (+)-norFFA (**C**) and (−)-norFFA (**D**)

Chemically, FFA used in the clinic is a racemic mixture, meaning that equal amounts of left-, and right-handed stereo-isomers (enantiomers) of the chiral molecule are present, i.e. (−)-FFA or levoFFA and (+)-FFA or dexFFA (Fig. 1). In addition, pharmacokinetic investigations have shown that FFA is substantially metabolized to norfenfluramine (norFFA), a *N*-dealkylated derivative of FFA resulting in circulating plasma levels that are similar to or greater than that of FFA itself, in human and animal models [13, 14]. Notably, circulating norFFA also consists of a racemic mixture of (−)-norFFA and (+)-norFFA (Fig. 1). As the various FFA and norFFA enantiomers are endowed with somewhat differing pharmacological profiles involving especially 5-HT, and type 1 sigma (σ1) receptors [13, 15], possibly resulting in a different antiepileptic activity, it is presently not known whether the efficacy of racemic FFA in the treatment of DS is due to a single enantiomer of FFA, or to both, and whether the norFFA enantiomers also contribute significantly.

Zebrafish are vertebrate models with genetic, physiological and CNS features that are highly conserved across vertebrates, including humans. Since zebrafish *scn1Lab* is evolutionarily close to the mammalian *SCN1A* gene [16], a zebrafish *scn1Lab double indemnity* (*didy*^*55*2^) mutant model has been used to find new medication to treat DS patients [17]. Homozygous *scn1Lab* mutants display spontaneously occurring seizures and brain epileptiform discharges, facilitating their use in phenotype-based screening projects [17].

In this study, the antiepileptic activity of the enantiomers of FFA and norFFA was explored using the zebrafish *scn1Lab*^*−*/*−*^ mutant model of DS in behavioral and electrophysiological assays. To validate the experimental conditions used, we explored the activity of a set of AEDs typically used in the fight against DS, including combination therapies. Finally, also the uptake of all four compounds in the larval zebrafish heads as a function of incubation time was assessed using Liquid Chromatography Mass Spectrometry (LC-MS).

## Materials and Methods

### Zebrafish Maintenance

Husbandry conditions of adult wild-type (AB-strain) and *scn1Lab* heterozygous mutant zebrafish (Danio rerio) were as described previously [18]. Fertilized eggs were collected following natural spawning. Then embryos and larvae were sorted and raised in embryo medium (0.3 × Danieau’s solution: 1.5 mM HEPES, pH 7.6, 17.4 mM NaCl, 0.21 mM KCl, 0.12 mM MgSO_4_, 0.18 mM Ca(NO_3_)_2_) in a Peltier-cooled incubator (IPP 260, Memmert, Schwabach, Germany) at 28 °C, using a 14/10 light/dark cycle. At 6 days post-fertilization (6 dpf) *scn1Lab*^*−*/*−*^ mutant larvae were selected by their darker appearance, lack of a swim bladder and slight curvature of the body, as performed before [18].

### Compound Preparation

Valproate (VPA), topiramate (TPM), stiripentol (STP), cannabidiol (CBD), clobazam (CLB), levetiracetam (LEV), carbamazepine (CBZ), and lamotrigine (LTG) were purchased from Sigma-Aldrich. Phenytoin (PHT) was from Acros Organics. (±)-Fenfluramine [(±)-FFA] was a gift from Prof. Berten Ceulemans (University of Antwerp, Belgium). The enantiomers of FFA and norfenfluramine (norFFA) were provided by Zogenix International (Emeryville, USA). Compounds were dissolved in dimethylsulfoxide (DMSO), and diluted in embryo medium to achieve a final DMSO concentration of 0.1% w/v. Embryo medium with 0.1% w/v DMSO served as a vehicle control (VHC).

### Toxicity Evaluation

To evaluate the maximum tolerated concentration (MTC) of the individual compounds, a dozen of WT zebrafish larvae (6 dpf) were individually incubated in single wells of a 96-well plate, and treated with two-fold serial dilutions of the compounds. After 22 h incubation under standard conditions (28 °C, 14/10 light/dark cycle), larvae were individually examined for their touch response, posture, edema, signs of necrosis, morphology, heartbeat rate, and swim bladder condition under the microscope. The MTC was defined as the highest concentration at which a compound did not exert any sign of toxicity in any of the larvae used.

### Locomotor Activity Measurement

WT and homozygous zebrafish larvae (*scn1Lab*^*−*/*−*^ mutants) (6 dpf) were individually positioned in single wells of a 96-well plate, and incubated in 100 μL VHC or VHC supplemented with compound or combined compounds for 22 h at 28 °C on a 14/10 h light/dark cycle. Then, the plates were placed immediately in an enclosed tracking device (ZebraBox Viewpoint, France), followed by a 30 min chamber habituation and 10 min recording. Locomotor activity over the total tracking period of 10 min was quantified by ZebraLab software (Software Viewpoint, France) using the lardist parameter (total distance in large movements) and 150 bkg (background) as a threshold, as reported previously by our group [18, 19]. For “small/large movement” and “inact/small movement”, a threshold was used of 6.4 and 3.3, respectively. Data were pooled from three or four independent experiments, with at least five larvae for each treatment and 20–30 larvae for each experimental condition, and expressed as “cm” per 100 s.

### Local Field Potential Recordings

WT and homozygous zebrafish larvae (*scn1Lab*^*−*/*−*^ mutants) (6 dpf) were treated as described above. After incubation, larvae were immobilized in 2% low-melting-point agarose (Invitrogen) at room temperature (RT) and the epileptiform activities were measured by noninvasive local field potential (LFP) recording from the skin above the optic tectum (midbrain). The single glass electrode filled with artificial cerebrospinal fluid (ACSF) (124 mM NaCl, 2 mM KCl, 2 mM MgSO_4_, 2 mM CaCl_2_, 1.25 mM KH_2_PO_4_, 26 mM NaHCO_3_, and 10 mM glucose) was positioned on the skin above the optic tectum. Each recording lasted for 10 min. Epileptiform activity was quantified by using Clampfit 10.2 software (Molecular Devices Corporation, USA60), as reported previously by our group [18, 19].

### Measurement of Compound Concentration in Heads

#### Extraction Procedure

WT and homozygous zebrafish larvae (*scn1Lab*^*−*/*−*^ mutants) (6 dpf) were treated as described above. After incubation with compounds, larvae were washed and euthanized by exposure to cold Milli-Q water. Then, the heads of larvae were carefully separated under the microscope, and five heads were transferred collectively into one 1.5 mL Eppendorf tube with acid-washed glass beads (diameter: 710–1180 µM, Sigma Aldrich) and 275 µL extraction medium (HPLC grade methanol, Sigma Aldrich). Next, the samples were homogenized by 10 min of ultrasonication (Diagenode Bioruptor Plus, Belgium) at 4 °C. The overall ultrasonication process encompassed 10 cycles of 30 s with pauses of 30 s in-between with high energy input [20]. After the subsequent centrifugation (14,500 g, 15 min), 200 µL of supernatant were collected from each tube, and stored in − 80 °C for further LC-MS processing.

#### HPLC Instrumentation and Quantification

Analyses were performed using an Infinity 1200 LC system (Agilent Technologies, Waldbronn, Germany) equipped with an autosampler, binary pump, and a thermostated column oven compartment. An YMC-Triart C18 (50 × 2.0 mm; *d*_p_ = 1.9 μm) column was utilized for the chromatographic separations at 40 °C. Samples were injected in a volume of 1 μL and the flow rate was set to 0.2 mL/min. The mobile phase consisted of 10 mM ammonium acetate in H_2_O: acetonitrile 75:25 (*v/v*) and separations were carried out isocratically. The elution time of (−)- and (+)-FFA was 4.1 min, while the elution time of (−)- and (+)-norFFA was 2.6 min. The LC instrument was hyphenated to a mass spectrometer (MS) with a triple quadrupole detector (API 3000, Applied Biosystems, Carlsbad, CA, USA) and equipped with an electrospray ionization source. The MS/MS analysis was conducted in multiple reaction mode (MRM) in positive mode. The MS settings were optimized by direct infusion of compound standards diluted in methanol. The optimal MS parameters are summarized in Table 1.

**Table 1:** Optimized mass settings for FFA and norFFA

Compound	Transition (Da)	DP (V)	FP (V)	EP (V)	CE (eV)	CXP (V)	NEB (psi)	CUR (psi)	CAD (psi)	Heater gas (L/min)	TEM (°C)	IS (V)
FFA	232 > 159	40	180	10	29	10	8	6	4	7	300	5500
norFFA	204 > 159	33	120	10	29	8	9	9	6	4	300	5500

*DP* declustering potential, *FP* focusing potential, *EP* entrance potential, *CE* the collision energy, and *CXP* the collision cell exit potential, *NEB* the nebulization gas, *CUR* the curtain gas, *CAD* the collision gas, *TEM* the temperature and *IS* the ionspray voltage

Quantification was performed through the use of a calibration curve created for each compound separately in blank matrix, which was derived from a pooled set of blank fish heads (n = 40). Calibrators consisted of 90 µL of blank matrix spiked with 10 µL of the corresponding standard dissolved in MeOH to obtain a concentration range varying between 0.025 μM to 7.5 μM for (+)-FFA, 0.02 μM to 5 μM for (−)-FFA, 0.05 μM to 10 μM for (+)-norFFA, and 0.02 μM to 10 μM for (−)-norFFA. Ranges consisted of at least 5 concentrations, each of which was analyzed in replicate (n = 5). A weighed least squares (WLS) regression model with 1/*x*^2^ weighing was utilized such that back-calculated concentrations did not deviate more than 15% from their nominal value. The final values of the regression coefficients (R^2^) were 0.993, 0.998, 0.992, and 0.990 for (+)-FFA, (−)-FFA, (+)-norFFA and (−)-norFFA, respectively.

The mean head weight (± S.D.) of a 6 dpf and 7 dpf zebrafish larva was 192 ± 10 µg and 196 ± 13 µg, respectively, as measured by weighing three batches of 50 fresh heads after removing excess water with filter paper. The final uptake was calculated according to the method reported by Copmans et al. [21], and expressed as amount/head weight (µg/g).

### Statistical Analysis

The locomotor activity data were analyzed by one-way ANOVA followed by Dunnett’s multiple comparison tests. Electrographic brain activity data were analyzed by Kruskal–Wallis testing with Dunn’s multiple comparisons. All test were performed in GraphPad Prism 8 software (GraphPad Software, Inc, USA). Significance was calculated only when compound treatment decreased the seizure activity. Significance levels: ^*^p ≤ 0.05, ^**^p ≤ 0.01, ^***^p ≤ 0.001, ^****^p ≤ 0.0001.

## Results

### ***Pharmacological Evaluation of the Zebrafish scn1Lab***^***−/−***^*** Mutant Model***

To validate the zebrafish *scn1Lab*^*−*/*−*^ mutant model and the experimental conditions used in this study, we first tested a series of antiepileptic drugs (AEDs) proposed by different treatment algorithms for DS [8–10, 22, 23], including valproate (VPA), racemic fenfluramine [( ±)-FFA], topiramate (TPM), stiripentol (STP), cannabidiol (CBD), clobazam (CLB) and levetiracetam (LEV). In addition, AEDs that should be avoided by DS patients, like carbamazepine (CBZ), phenytoin (PHT) and lamotrigine (LGT) were examined as they target the sodium channel resulting in seizure aggravation [6, 24].

Various concentrations of the AEDs were examined for their adverse effects, allowing a maximum tolerated concentration (MTC) to be determined. MTC is defined as the highest concentration at which the compound did not exert any sign of toxicity in any of the larvae tested. MTC values were determined as 1 mM for VPA, 50 µM for ( ±)-FFA, 200 µM for TPM, 50 µM for STP, 6.25 µM for CBD, 100 µM for CLB, 10 mM for LEV, 50 µM for CBZ, 100 µM for PHT, and 100 µM for LTG. By using these maximal concentrations for all further investigations, we sought to reduce the risk of false positive results to a minimum, which is particularly critical for locomotor activity measurements.

**Fig. 2:**
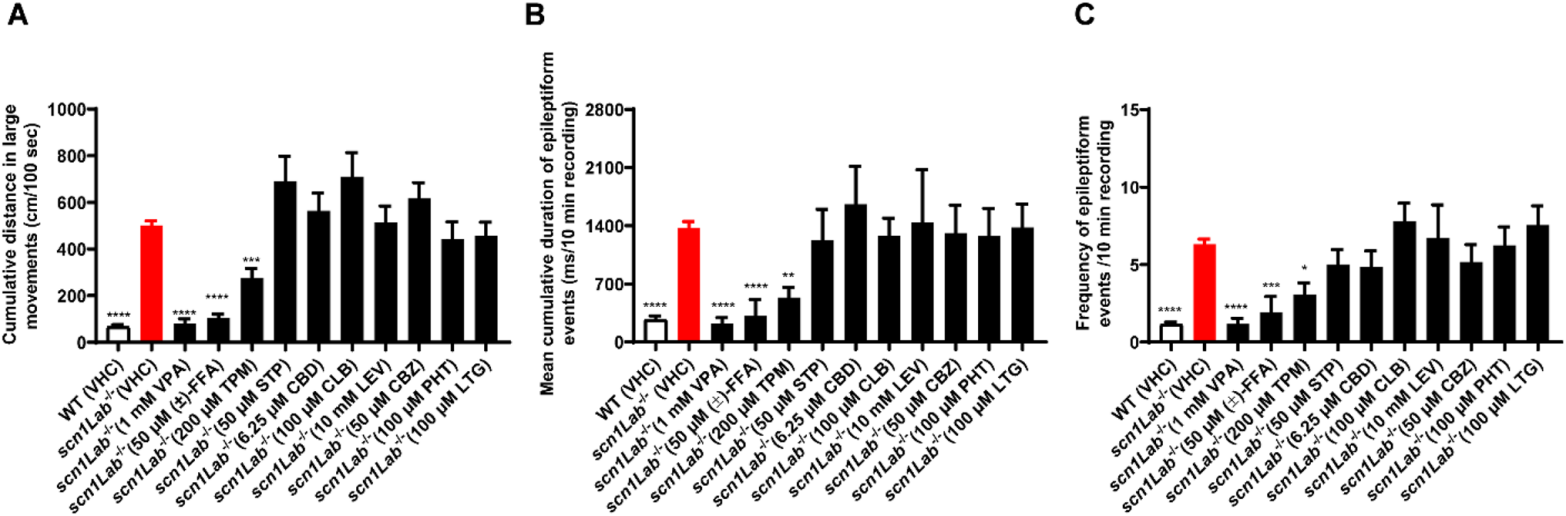
Behavioral (**A**) and electrophysiological (**B**, **C**) antiepileptic activity of valproate (VPA), (±) fenfluramine [(±)-FFA], topiramate (TPM), stiripentol (STP), cannabidiol (CBD), clobazam (CLB), levetiracetam (LEV), carbamazepine (CBZ), phenytoin (PHT), and lamotrigine (LTG) in the zebrafish *scn1Lab*^*−*/*−*^ mutant model. (A) Locomotor activity of larvae pre-exposed to antiepileptic drugs (AEDs) for 22 h. Data were assessed over the total tracking period of 10 min and expressed as cm/100 s. Results were pooled from 3–4 independent experiments, with 207 larvae for each of the VHC-treated groups, and 22–31 larvae for each AED-treated group. (**B**, **C**) Noninvasive local field potential (LFP) recordings from the optic tectum of larvae pre-exposed to antiepileptic drugs (AEDs) for 22 h. Epileptiform discharges are quantified by the cumulative duration (mean ± SEM) (**B**) and frequency (mean ± SEM) (**C**) of events per 10-min recording. With 72 larvae for the VHC-treated group, 10–15 larvae for each AED-treated group. Statistical analysis: one-way ANOVA with Dunnett’s multiple comparison test (locomotor assay), Kruskal–Wallis testing with Dunn’s multiple comparisons (LFP measurements). Significance levels: ^*^p ≤ 0.05, ^**^p ≤ 0.01, ^***^p ≤ 0.001, ^****^p ≤ 0.0001. *WT* wide type, *VHC* vehicle

Next, the effect of each of these AEDs as a single treatment on locomotor and brain activities was evaluated by behavioral and electrophysiological assays using the zebrafish *scn1Lab*^*−*/*−*^ mutant model. As shown in Fig. 2, VPA elicited a complete rescue of the epileptiform locomotor activity (Fig. 2A, P ≤ 0.0001) and epileptiform brain discharges of the mutant larvae, as monitored by measuring the cumulative duration (Fig. 2B, P ≤ 0.0001) and frequency (Fig. 2C, P ≤ 0.0001).

Of interest, VPA rarely provides adequate seizure control in DS patients, so that it requires the addition of others AEDs as second-line therapies [10], whereas in our hands VPA fully corrected the seizure phenotype of the *scn1Lab*^*−*/*−*^mutants. In the clinic, however, VPA shows dose-limiting side-effects after prolonged use like fatigue, hair loss and hyperammonemia amongst others [25]. Possibly the relative short treatment of zebrafish used in this study allowed to use an immersion concentration that exceeds the corresponding clinical dose of VPA, resulting in an enhanced efficacy.

Similarly to VPA, (±)-FFA (Fig. 2A, P ≤ 0.0001) and TPM (Fig. 2A, P ≤ 0.001) not only significantly counteracted the increased locomotor activity observed in *scn1Lab*^*−*/*−*^ mutants, but also dramatically attenuated the frequency of epileptiform events (Fig. 2C, P ≤ 0.001 and P ≤ 0.05, respectively), resulting in a decrease of cumulative duration (Fig. 2B, P ≤ 0.0001 for ( ±)-FFA, P ≤ 0.01 for TPM).

By contrast, STP, CBD, CLB and LEV failed to rescue both the hyper-locomotor activity and brain epileptic discharges of *scn1Lab*^*−*/*−*^ mutants (Fig. 2). Also, as expected, no inhibitory effects could be observed with CBZ, PHT and LTG treatments (Fig. 2).

**Fig. 3:**
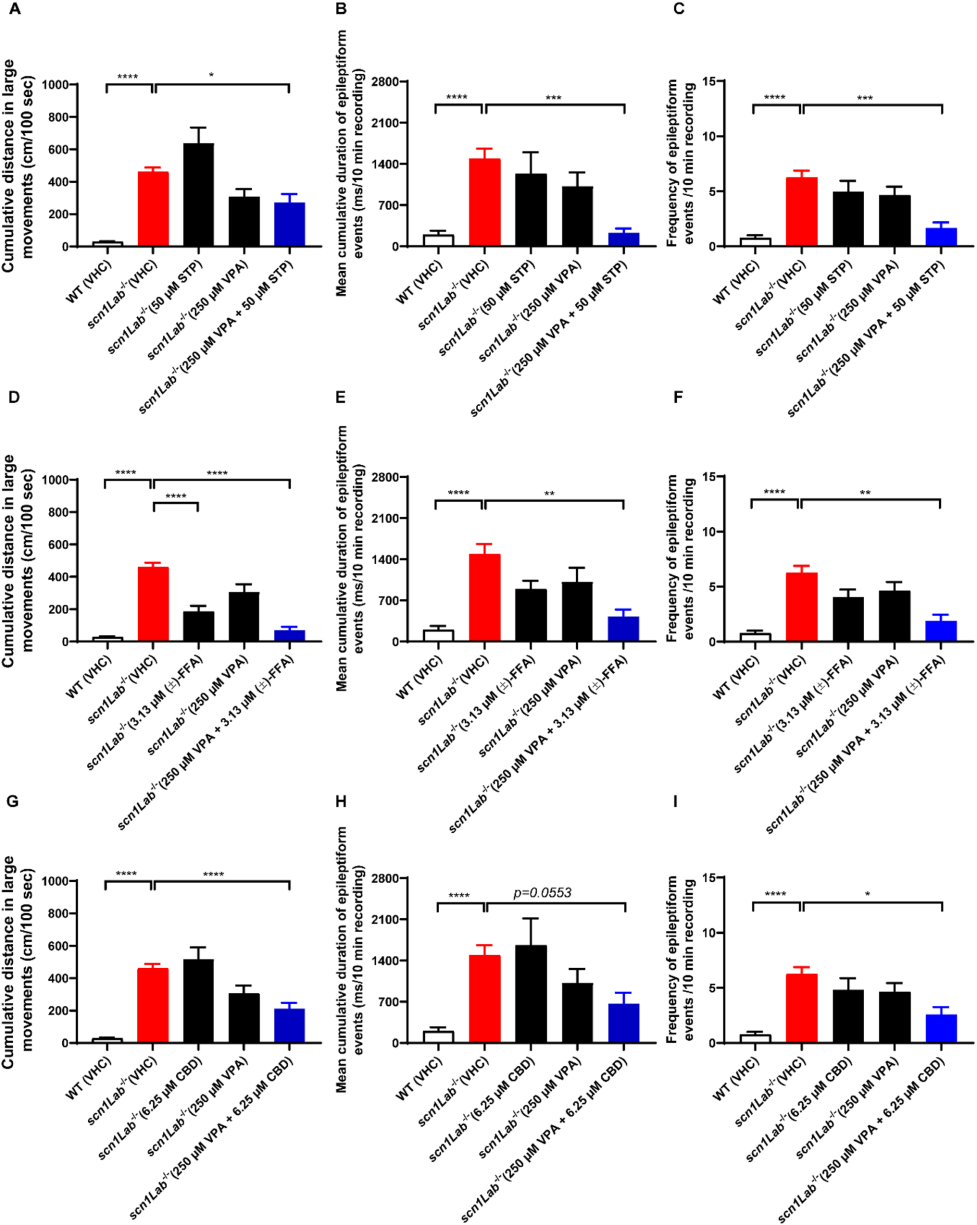
Behavioral (**A**, **D**, **G**) and electrophysiological (**B**, **C**, **E**, **F**, **H**, **I**) antiepileptic activity of combination treatment (colored in blue) of valproate (VPA) with stiripentol (STP) (**A**–**C**), (±) fenfluramine [(±)-FFA] (**D**–**F**), and cannabidiol (CBD) (**G**–**I**) in the zebrafish *scn1Lab*^*−/−*^ mutant model. (**A**, **D**, **G**) Locomotor activity of larvae pre-exposed to antiepileptic drugs (AEDs) for 22 h. Data were assessed over the total tracking period of 10 min and expressed as cm/100 s. Results were pooled from 3–4 independent experiments, with 120 larvae for each of the VHC-treated groups, and 20–30 larvae for each AED-treated group. (**B**, **C**, **E**, **F**, **H**, **I**) Noninvasive local field potential (LFP) recordings from the optic tectum of larvae pre-exposed to antiepileptic drugs (AEDs) for 22 h. Epileptiform discharges are quantified by the cumulative duration (mean ± SEM) (**B**, **E**, **H**) and frequency (mean ± SEM) (**C**, **F**, **I**) of events per 10-min recording. With 23 larvae for the VHC-treated group, 12–14 larvae for each AED-treated group. Statistical analysis: one-way ANOVA with Dunnett’s multiple comparison test (locomotor assay), Kruskal–Wallis testing with Dunn’s multiple comparisons (LFP measurements). Significance levels: ^*^p ≤ 0.05, ^**^p ≤ 0.01, ^***^p ≤ 0.001, ^****^p ≤ 0.0001. *WT* wild type, *VHC* vehicle

As STP, CBD and (±)-FFA are typically combined in the clinic with VPA for second line treatment [10], we further validated the *scn1Lab*^*−*/*−*^ mutant model by exploring the activity of this combination treatment. Since VPA as a single treatment at 1 mM completely reduced the read-outs to the level exhibited by AB control larvae, we modified its concentration to 250 µM. This concentration exerted a limited and statistically non-significant effect on the locomotor activity and epileptiform brain discharges of the mutant larvae (Fig. 3).

Significantly, the combination of VPA (250 µM) and STP (50 µM) turned out to effectively alter the locomotor activity and decrease the epileptiform discharges of the mutant larvae, whereas the single treatments were not active (Fig. 3A–C).

Furthermore, investigating the combination outcome of VPA (250 µM) and (±)-FFA, the concentration of the latter compound was reduced from 50 µM (MTC) to 3.13 µM. This concentration continued to induce a significant effect on the locomotor activity but not on the epileptiform brain discharges of the mutant larvae (Fig. 3D-F). The combined compounds diminished the locomotor activity of mutant larvae treated with 3.13 µM (±)-FFA (single treatment) by more than half on average, although the difference observed was statistically not significant (Fig. 3D). The LFP results further show that the combination of VPA and (±)-FFA was highly effective in reducing the epileptiform discharges of the mutant larvae, whereas the single treatments were not (Fig. 3E, F).

Finally, when VPA (250 µM) was combined with CBD (6.25 µM), the VPA + CBD-treatment showed a clear effect on the locomotor and LFP results obtained with the mutant larvae as compared to the single treatments that were not active (Fig. 3G-I).

As far as alternatives for second line treatment are concerned [10], i.e. TPM and CLB, only the former exhibited a pronounced therapeutic activity in the *scn1Lab*^*−*/*−*^mutant model. Noticeably, CLB was previously proven to be ineffective in the DS zebrafish mutant model [26]. Although CLB has also been suggested as a first-line drug by the North American consensus panel, it typically only displays efficacy in DS patients when combined with VPA and STP [9]. Whether CLB therefore classifies as a true false negative in the *scn1Lab*^*−*/*−*^mutant model is yet to be investigated into more detail, for instance by exploring its additional activity in combination with VPA and STP, as suggested by Cross et al. [10].

LEV has been categorized as a third-line drug for DS by the North American consensus panel [9], but is not mentioned in the treatment options by Cross et al. [10]. As a matter of fact, there is limited clinical evidence regarding its efficacy in the clinic, with retrospective studies demonstrating low responder rates in DS patients [23]. Significantly, the compound also failed to suppress the seizure-like activity of *scn1Lab*^*−*/*−*^ mutants in this study, as shown before [17].

### Determination of the Time-Dependent Concentration of Enantiomers of FFA and norFFA in Zebrafish Head

**Fig. 4:**
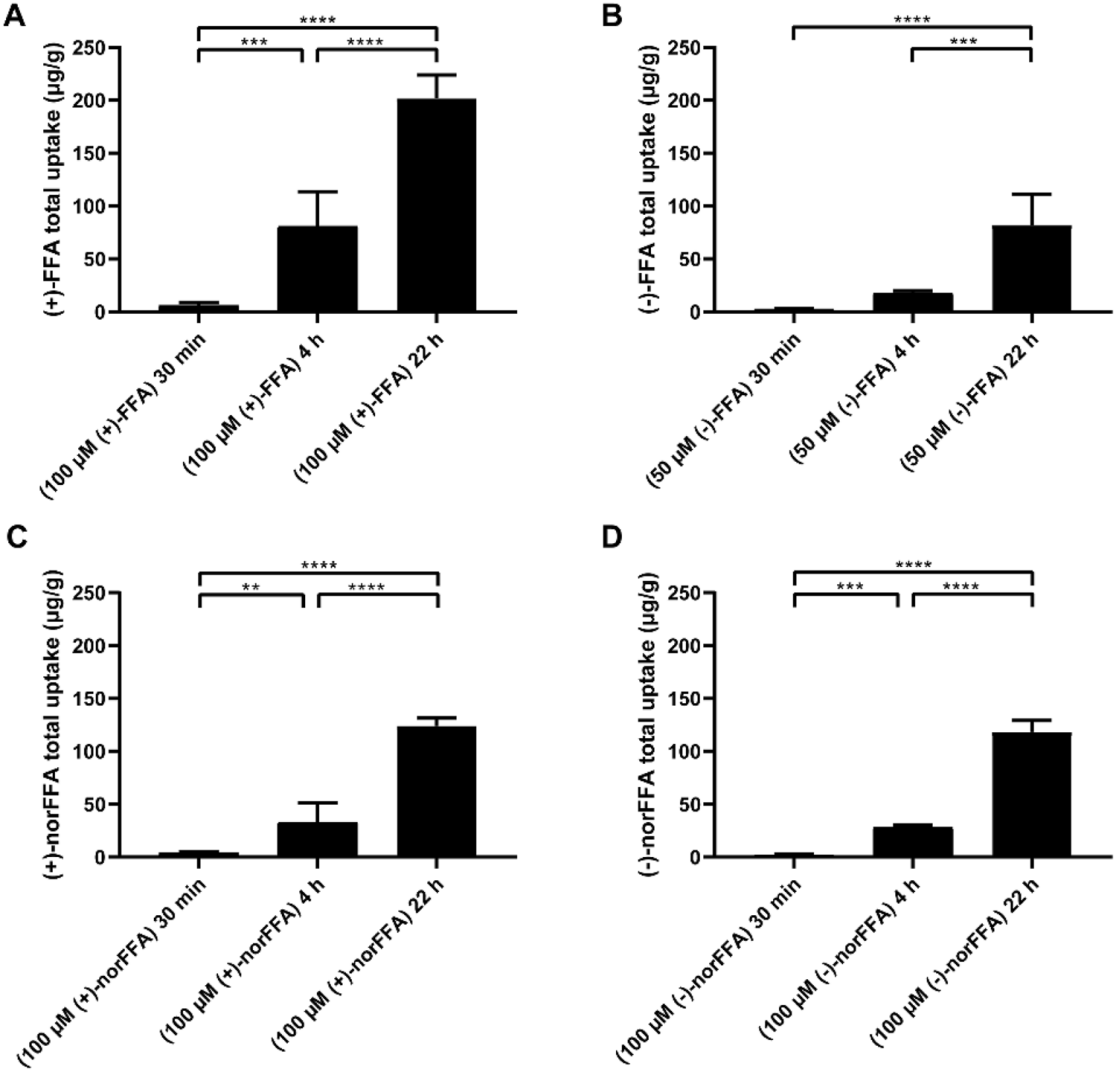
The total uptake of (+)-FFA (**A**), (−)-FFA (**B**), (+)-norFFA (**C**) and (−)-norFFA (**D**) in the larvae head after different exposure times (30 min, 4 h and 22 h). The concentration in the head of larvae pre-exposed to (+)-FFA (A), (−)-FFA (B), (+)-norFFA (**C**) and (−)-norFFA (**D**) at their respective MTCs for 30 min, 4 h and 22 h, separately, and total uptake of compound = compound concentration/head weight. With 5 larvae heads for each sample, and 5 replicates for a total of 25 larvae heads per treatment. Statistical analysis: one-way ANOVA with Dunnett’s multiple comparison test. Significance levels: ^*^p ≤ 0.05, ^**^p ≤ 0.01, ^***^p ≤ 0.001, ^****^p ≤ 0.0001. *WT* wild type, *VHC* vehicle, *FFA* fenfluramine, *norFFA* norfenfluramine

In order to investigate the uptake kinetics of the enantiomers of FFA and norFFA in larval zebrafish heads, we immersed larvae in solutions of the individual compounds at their MTC for 30 min, 4 h and 22 h. Next, the compounds present in extracts of the heads were quantified by LC-MS analysis. The data depicted in Fig. 4 show that the total uptake (amount of compound/head weight) of each enantiomer of FFA and norFFA increased in a time-dependent fashion.

A somewhat similar uptake was observed for the (+)-norFFA and (−)-norFFA enantiomers, implying that the levo/dextrotation of the structure did not dramatically affect the uptake. Conversely, a direct comparison between (+)-FFA and its (−)-enantiomer is hard to draw as different immersion concentrations were used due to the different MTCs of the respective compounds.

### ***Antiepileptic Activity of Enantiomers of FFA and norFFA in the Zebrafish scn1Lab***^***−/−***^*** Mutant Model***

**Fig. 5:**
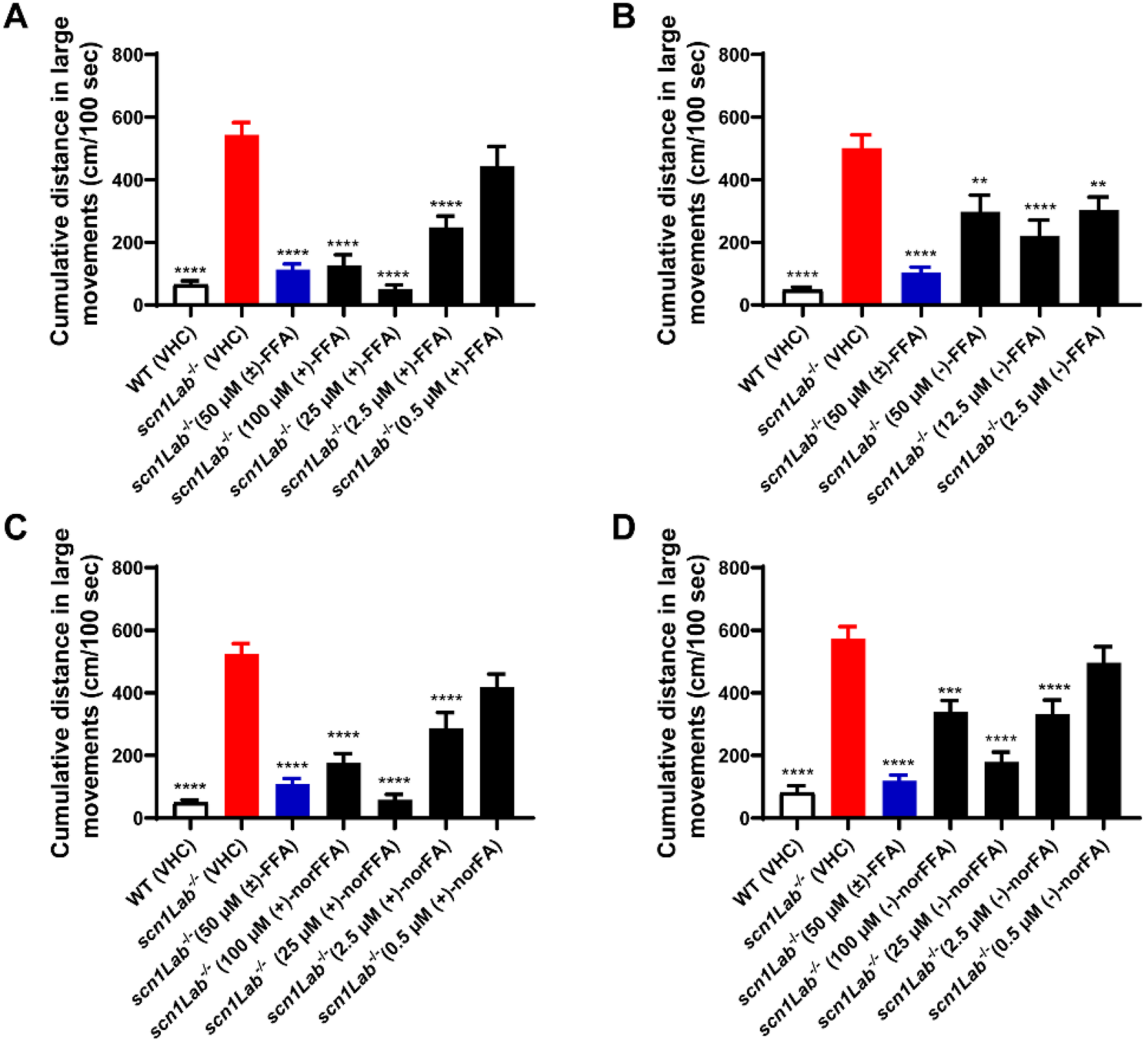
Behavioral antiepileptic activity of (+)-FFA (**A**), (−)-FFA (**B**), (+)-norFFA (**C**) and (−)-norFFA (**D**) in the zebrafish *scn1Lab*^*−*/*−*^ mutant model, and ( ±)-FFA (colored in blue, **A**–**D**), used as a positive control. (**A**–**D**) Locomotor activity of larvae pre-exposed to different concentration of enantiomers of FFA and norFFA for 22 h. Data were assessed over the total tracking period of 10 min and expressed as cm/100 s. Results were pooled from 3–4 independent experiments, with 67–75 larvae for each VHC-treated group, and 21–28 larvae for each compound-treated group. Statistical analysis: one-way ANOVA with Dunnett’s multiple comparison test. Significance levels: ^*^p ≤ 0.05, ^**^p ≤ 0.01, ^***^p ≤ 0.001, ^****^p ≤ 0.0001. *WT* wild type, *VHC* vehicle, *FFA* fenfluramine, *norFFA* norfenfluramine

As uptake of compounds in larval heads was maximal after 22 h, we proceeded to use this prolonged incubation condition to explore the pharmacological activity of the FFA and norFFA enantiomers, as performed previously [18, 19]. To investigate the potency of the compounds to prevent the epileptiform activity exhibited by the *scn1Lab*^*−*/*−*^ mutants, they were first examined by a behavioral assay, at a wide range of concentrations. As illustrated in Fig. 5, all enantiomers of FFA and norFFA effectively counteracted abnormal locomotor activity of mutant larvae at their MTC, 1/4 MTC and 1/40 MTC [1/20 MTC for (−)-FFA], whereas lower concentrations were not active. In addition, all drugs displayed their maximum effects at their respective 1/4 MTCs (Fig. 5, P ≤ 0.0001).

**Fig. 6:**
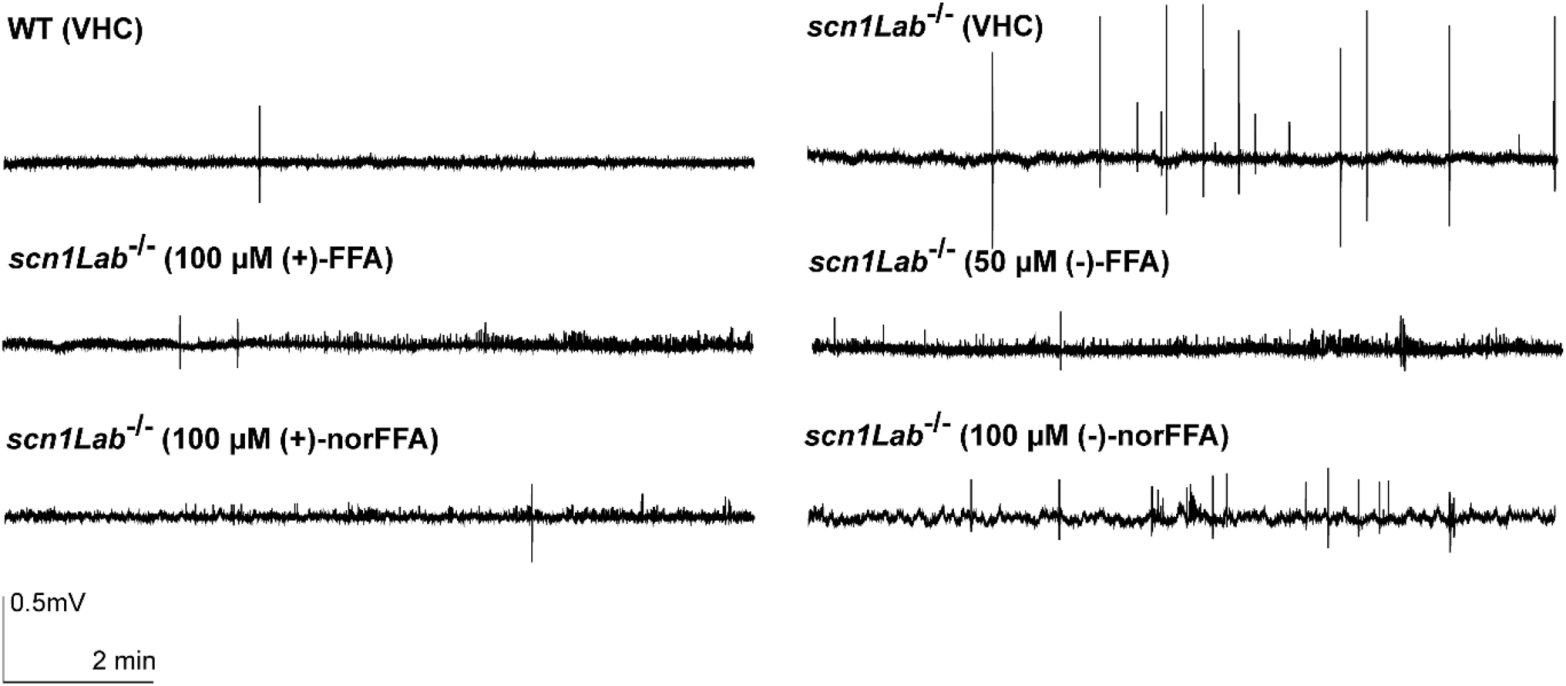
Representative local field potential recordings. Ten-min noninvasive local field potential (LFP) recordings from the optic tectum of larvae pre-exposed to (+)-FFA, (−)-FFA, (+)-norFFA and (−)-norFFA for 22 h. *WT* wild type, *VHC* vehicle, *FFA* fenfluramine, *norFFA* norfenfluramine

**Fig. 7:**
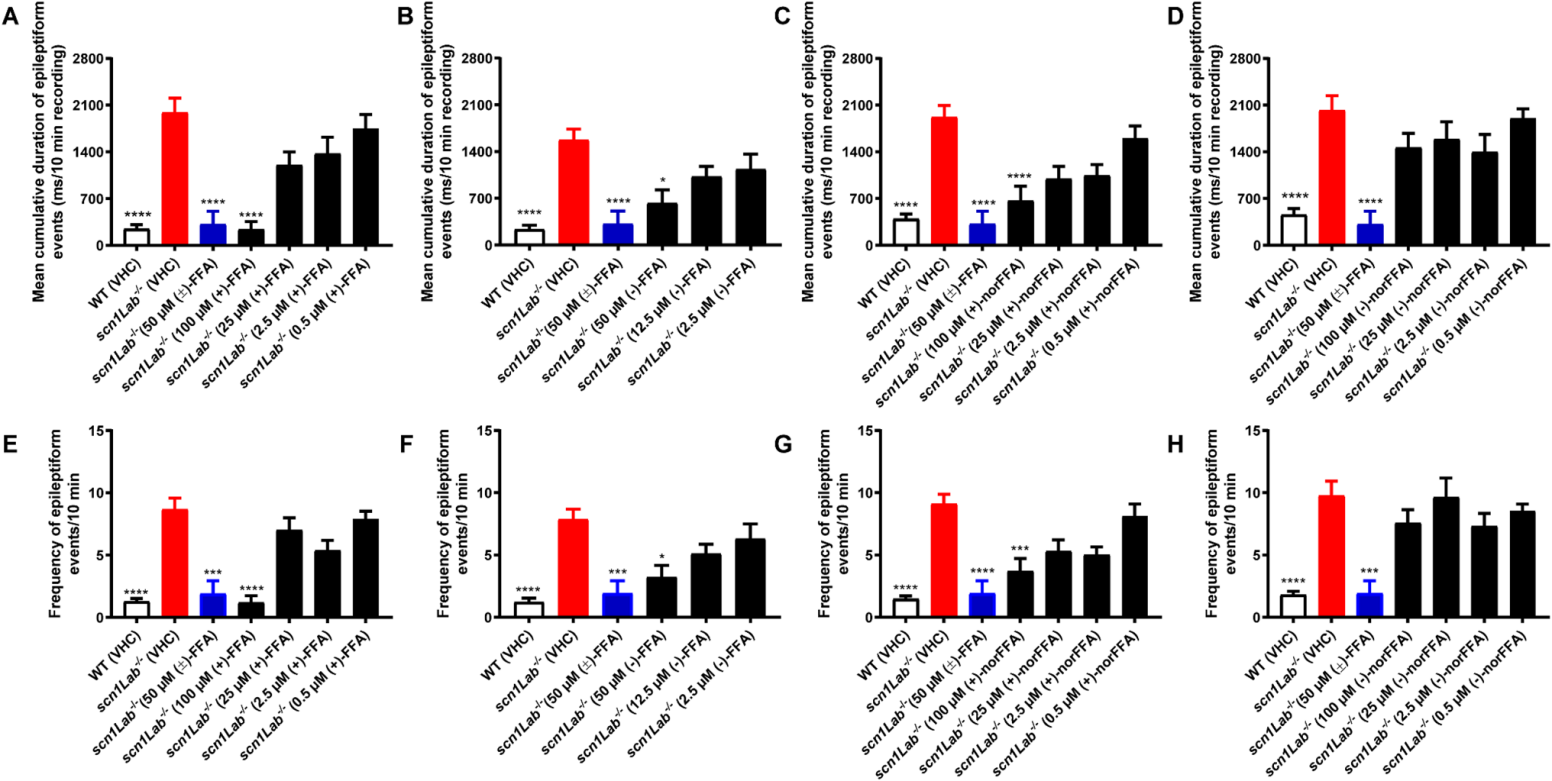
Electrophysiological antiepileptic activity of (+)-FFA (**A**, **E**), (−)-FFA (**B**, **F**), (+)-norFFA (**C**, **G**) and (−)-norFFA (**D**, **H**) in the *scn1Lab*^*−*/*−*^ mutant model, and ( ±)-FFA (colored in blue, **A**–**H**) used as a positive control. (**A**–**H**) Noninvasive local field potential (LFP) recordings from the optic tectum of larvae pre-exposed to (+)-FFA, (−)-FFA, (+)-norFFA and (−)-norFFA for 22 h. Epileptiform discharges are quantified by the cumulative duration (mean ± SEM) (**A**–**D**) and frequency (mean ± SEM) (**E**–**H**) of events per 10-min recording. With 27–45 larvae for each VHC-treated group, and 10–16 larvae for each compound-treated group. Statistical analysis: Kruskal–Wallis testing with Dunn’s multiple comparisons. Significance levels: ^*^p ≤ 0.05, ^**^p ≤ 0.01, ^***^p ≤ 0.001, ^****^p ≤ 0.0001. *WT* wild type, *VHC* vehicle, *FFA* fenfluramine, *norFFA* norfenfluramine

Subsequently, we examined the effect of the compounds on the epileptiform discharges of the mutant larvae by recording local field potentials (LFP) of the brain. Representative traces of brain activity during the recordings are shown in Fig. 6. As depicted in Fig. 7, all compounds except for (−)-norFFA significantly reduced the frequency and cumulative duration of the epileptiform events, at least at their MTC. The results therefore confirm most of the results obtained using the locomotor assay, although some concentration-related discrepancies exist, especially in the case of (−)-norFFA. A different outcome between the two assays has also been reported by others [17, 19, 27], possibly the result of some extra peripheral off-target effects of compounds affecting the locomotor read-out.

## Discussion

Overall, our results mirror well the order of multiple treatment options proposed by the updated current practice in management of DS [10]. Notably, a similar validation of the *scn1Lab*^*−*/*−*^ mutant zebrafish DS model was previously performed, using a shorter incubation protocol than present in this study [17, 28]. However, to our knowledge, this is the first study that shows the activity of combined AED therapy, thereby further corroborating the zebrafish DS model. Moreover, the outcome of this study seems to indicate that (+)-FFA and especially (+)-norFFA exhibited inhibitory profiles that were more consistent and concentration-dependent than the (−)-enantiomers, for both locomotor and LFP read-outs.

Surprisingly, a substantial increase in concentrations could still be observed in the 4–22 h time window. To the best of our knowledge, this study is the first to quantify the uptake of any drug compound in heads of zebrafish larvae as a function of time, and consequently it is not possible to conclude whether FFA and norFFA are actually unique in this respect. However, a certain parallel is apparent with the clinical condition, where accumulation of (+)-FFA and (+)-norFFA in humans typically follows a step-wise increase to steady state concentrations after long-term repetitive drug administration [29].

Other studies reported the uptake of compounds in heads and brain of larval zebrafish after 1 h incubations [20, 30]. Of interest, when measuring head uptake of haloperidol (clog P value: 4.3) or diphenhydramine (clog P value: 3.3) after incubating 5 dpf zebrafish larvae for 1 h with 15 µg/mL of the compounds, the recovered concentrations were 21.6 µg/g and 115.5 µg/g, respectively [30]. As the lipophilicity of compounds is a crucial determinant for the uptake in body and brain tissue [20, 31], and the clog P values of FFA and norFFA are 3.47 and 2.68, respectively, the aforementioned data are in line with the results obtained after 1–4 h incubations in this study. Significantly, when comparing (+)-FFA with the less lipophilic (+)-norFFA, the data clearly show that the nor-metabolite of FFA is taken up less than the parent compound.

Obviously, the activity of the compounds is determined by their relative uptake in brain tissue in combination with their underlying molecular mechanisms. Of importance, an increasing number of reports has indicated that low 5-HT (serotonin) brain levels are involved in epileptogenesis and/or seizure propagation [32, 33], and a 5-HT deficit was also reported in the heads of homozygous *scn1Lab*^*−*/*−*^ mutants [19]. Significantly, as shown in Table 2, both enantiomers of FFA and norFFA are potent substrates for 5-HT transporter proteins, with EC_50_ values ranging from 52 to 287 nM. Other effects of the enantiomers relate to potent serotonin uptake inhibition and agonistic effects on 5-HT_2_ subtype receptors (Table 2) [13, 34]. Effects on dopamine and norepinephrine uptake and release have also been documented, although a wide range of potencies were found for the different FFA and norFFA enantiomers (Table 2). In addition, the (+)-enantiomers of FFA and norFFA can diminish glutamatergic N-methyl-D-aspartate (NMDA) neurotransmission through disrupting its association with type 1 sigma (σ1) receptors, thereby acting as highly potent σ1R antagonists [15]. Unfortunately, the activity of the individual enantiomers of FFA and its metabolites were not examined in the latter study. Significantly, using similar conditions as in this study (i.e. 25 µM, 22 h incubation), the racemic mixture (±)-FFA was found to exert its anti-seizure activity in DS zebrafish mainly through its modulation of 5-HT_2C_-R, 5-HT_1D_-R, sigma-1-R and possibly 5-HT_2A_-R [18], thereby confirming the aforementioned data obtained with mammalian cell-based assays.

**Table 2:** Potency of enantiomers of FFA and norFFA

Compound	EC_50_ values (nM) for release			K_i_ values (nM) for uptake inhibition			K_act_ values (nM) for 5-HT_2_ receptor subtypes		
	DA	NE	5-HT	DA	NE	5-HT	5-HT_2A_	5-HT_2B_	5-HT_2C_
( +)-FFA	> 10,000	302 ± 20	51.7 ± 6.1	> 20,000	1286 ± 52	150 ± 5	> 10,000	379 ± 70	362 ± 64
( −)-FFA	> 10,000	> 10,000	147 ± 19	> 20,000	7187 ± 559	714 ± 31	5279 ± 587	1248 ± 252	360 ± 91
( +)-norFFA	924 ± 112	72.7 ± 5.4	59.3 ± 2.4	2312 ± 87	205 ± 19	214 ± 9	630 ± 141	18.4 ± 5.3	13 ± 2.4
( −)-norFFA	> 10,000	474 ± 40	287 ± 14	19,194 ± 1,048	2052 ± 297	1175 ± 89	1565 ± 190	357 ± 105	18 ± 3.5

Adapted from Rothman et al. [13, 34]

The data available therefore show that the enantiomers of FFA and norFFA possess somewhat different pharmacological potencies on a subset of receptors that have been implicated in their anti-epileptic activity. However, in view of the larval head concentrations of the individual compounds found in this study, one would not anticipate any major difference in outcome for both locomotor and LFP read-outs. Moreover, as the pharmacological fingerprint of (−)-norFFA is not substantially different from the one of (−)-FFA, it is rather surprising that the former compound exerted a less conclusive inhibitory activity in the DS zebrafish model. Evidently, the slightly conflicting results might be explained by enantiomers’ relative affinities for zebrafish proteins differing to those for their mammalian counterparts. However, the mechanism-of-action of the antiepileptic effect of FFA is multi-dimensional, also involving σ1-receptors and possibly other targets, further complicating the interpretation of the finding. Clearly more investigations are needed to better understand the relationship between the pharmacology of the compounds and their respective antiepileptic activities against DS.

Taken together, our study is the first to validate the *scn1Lab*^*−*/*−*^ mutant model by using a combined treatment of AEDs, further supporting the application of the zebrafish-based model as a rapid screening platform to find precision medicine for DS, and possibly for other difficult-to-treat epilepsies. In addition, our results show that (+)-FFA, (−)-FFA and (+)-norFFA displayed significant antiepileptic effects in the preclinical model, and thus can be considered as compounds actively contributing to the clinical efficacy of FFA. In case of (−)-norFFA, the results were less conclusive. Whether this inconsistency is related to a different pharmacological fingerprint is presently unexplored and warrants further investigation.

## Data Availability

All data generated or analyzed during this study are included in this published article.
